# Supplementary material for: Chromothripsis during telomere crisis is independent of NHEJ, and consistent with a replicative origin
Source: Genome Res. 2019 May;29(5):737–49. doi: 10.1101/gr.240705.118 (PMC6499312; doi:10.1101/gr.240705.118)
Supplement: Supplemental Material [file supp_gr.240705.118_Supplemental_file_1.zip › contigs/annotated_contigs/DB81/contig.2.DB81_length_656_mean_cov_13.0365853659.docx]

**DB81_length_656_mean_cov_13.0365853659**

```
AATATATAATTTGCAATGTTCATGGCAAGACATTTTAACCATACAAGAGAAAAAAAGGTCAAAAATTCCCTTTTCTCACCCCTTCTACT
 >chr7:18596965-18597291 - E=2e-184
ACTACTACTAGTAGAAATCCCTAGTAACAGATTCTGTGTATTCTCCATAAAGTCTATCTTTCATGAAATATTATGTTTATATCTCTATA

TATTATAGATGAAACAGATAACATAACTAAAATAAAGAACACACATAACAATCCCATTTTTATTTACATTTGTAATAAAGAAAACTTAG

GGAGAGGTGACTTTAAAGTATACCAGGAGGATTAAGGTTAGATATAAAAAAAAAAAAAA|CT|CACACCCATATAAGACGGCGAACTTA
                                                              >chr7:18587543-18587871 -
ATCTGTAAATGTTGTGTGTGGTGTTCCGACTTCACTGACCAGCAGTCAGTCTCTGACTCTCTGCCTCTCCTCAGGTCTCCCTATTCACA
 E=1e-185
GAGACACAAGAATATTGAAATTAGGGCAATTAATAACCCTACAATGGCCTCTAAGTGTTCAAGTGAAAGAGAAAGAGTTGCACATCTCT

CTTTCAATCAAGAGCTAGAAATGATTAAGCTGAGTTAGGAAGGCATGTTGACAGCCAAGACAGGTGGAGAGCAAGGCCTCTTGAACCAA

ACCACAAAATTGTGAATGCAAATGGAGGGAGGGAG
```
